# The therapeutic effect of mesenchymal stem cells in diabetic kidney disease

**DOI:** 10.1007/s00109-024-02432-w

**Published:** 2024-02-29

**Authors:** Umm E. Habiba, Nasar Khan, David Lawrence Greene, Sabiha Shamim, Amna Umer

**Affiliations:** 1 Pak-American Hospital Pvt. Ltd, Jahangir Multiplex, Peshawar Road, Sector H-13, Islamabad, 44000 Pakistan; 2 R3 Medical Research LLC, 10045 East Dynamite Boulevard Suite 260, Scottsdale, AZ 85262 USA; 3 Bello Bio Labs and Therapeutics (SMC) Pvt. Ltd., Jahangir Multiplex, Peshawar Road, Sector H-13, Islamabad, 44000 Pakistan

**Keywords:** Diabetic kidney disease, Regenerative medicine, Stem cell therapy, Mesenchymal stem cell, Clinical trials

## Abstract

**Abstract:**

Diabetes mellitus (DM) often causes chronic kidney damage despite best medical practices. Diabetic kidney disease (DKD) arises from a complex interaction of factors within the kidney and the whole body. Targeting specific disease-causing agents using drugs has not been effective in treating DKD. However, stem cell therapies offer a promising alternative by addressing multiple disease pathways and promoting kidney regeneration. Mesenchymal stem cells (MSCs) offer great promise due to their superior accessibility ratio from adult tissues and remarkable modes of action, such as the production of paracrine anti-inflammatory and cytoprotective substances. This review critically evaluates the development of MSC treatment for DKD as it moves closer to clinical application. Results from animal models suggest that systemic MSC infusion may positively impact DKD progression. However, few registered and completed clinical trials exist, and whether the treatments are effective in humans is still being determined. Significant knowledge gaps and research opportunities exist, including establishing the ideal source, dose, and timing of MSC delivery, better understanding of in vivo mechanisms, and developing quantitative indicators to obtain a more significant therapeutic response. This paper reviews recent literature on using MSCs in preclinical and clinical trials in DKD. Potent biomarkers related to DKD are also highlighted, which may help better understand MSCs’ action in this disease progression.

**Key messages:**

- Mesenchymal stem cells have anti-inflammatory and paracrine effects in diabetic kidney disease.
- Mesenchymal stem cells alleviate in animal models having diabetic kidney disease.
- Mesenchymal stem cells possess promise for the treatment of diabetic kidney disease.

## Introduction

Diabetes and chronic kidney disease (CKD) patients comprise a distinct subpopulation of people with diabetic kidney disease (DKD), which can be recognized by amplified excretion of albumin protein in the urine or lesser glomerular filtration rate (GFR), or maybe both [1]. It ultimately disturbs the normal process of eliminating unwanted products and fluids from the human body and intrudes on kidney function. The International Diabetes Federation (IDF) claims that 40% of patients with diabetes experience kidney failure in its most advanced stages. Additionally, diabetes mellitus and hypertension are critical indicators or sometimes frame 80% of end-stage renal failure cases (ESRF) [2].

## Clinical signs and symptoms

Albuminuria, or the removal of albumin in the urine, increased weight and inflammation of the legs and ankles; repeated nighttime urination, morning dizziness and illness, and anemia; and raised blood pressure are all indications of DKD in humans [3]. Type 2 diabetes mellitus (T2DM) patients are 40% more likely than type 1 diabetes patients to suffer DKD. DKD is the main factor causing end-stage renal disease, i.e., CKD and ESRD [4]. The uneven increase in low- to middle-socioeconomic nations and an underappreciated global illness burden is leading to a consistent rise in the prevalence of DKD [5]. Additionally, DKD is linked to a high death rate. Diabetes patients with kidney illness had a 31.1% higher mortality risk, which rose with the severity of the condition [6, 7]. Early DKD patients also had a greater mortality risk [8]. DKD also places a significant financial and societal strain [9]. DKD is often detected much later in a person until it manifests significant difficulties [10–12]. Lack of information and inconsistent screening are the main barriers to early diagnosis [13, 14]. Early diagnosis is a practical way to lessen the financial burden of DKD.

## DKD risk factors

### Escalated albuminuria

**Fig. 1:**
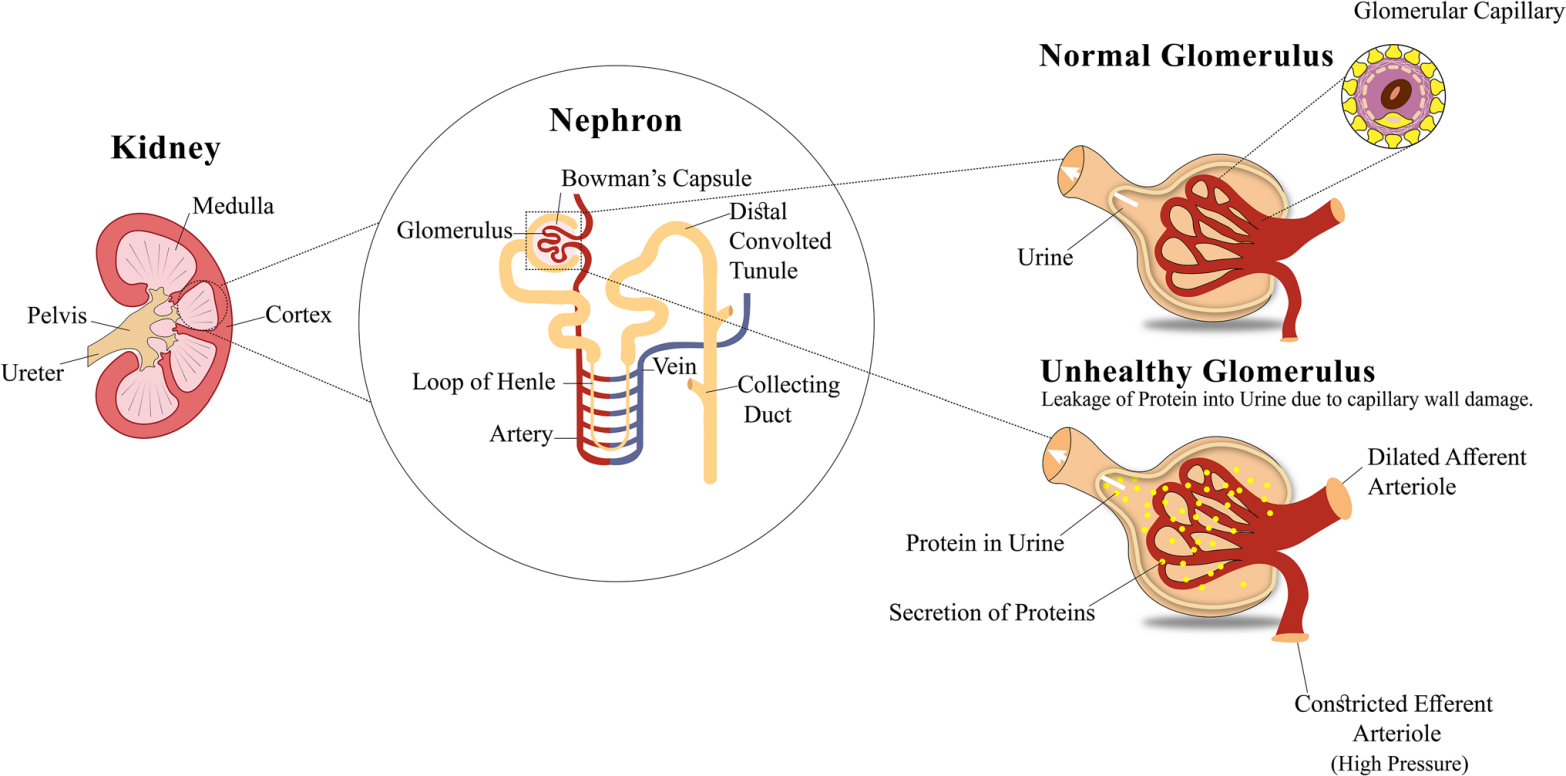
Diabetic kidney disease, kidney disease influenced by diabetes. Comprehensive anatomy of the glomerulus elucidates that in a healthy glomerulus, the capillary keeps the protein molecules in the blood, and routine urine will be passed out from the body. Meanwhile, in an unhealthy glomerulus, the glomerulus capillary wall is damaged, which results in the spilling of protein molecules into the urine. The unhealthy glomerulus will lead to escalated emission of albumin protein in the urine, causing DKD

Kidney disease progression is caused by the high emission of albumin in the urine. Microalbuminuria (30–300 mg g^−1^) or macroalbuminuria (> 300 mg g^−1^) in urine is described by increased emission of albumin creatinine [15]. Both micro- and macroalbuminuria are markers of kidney function impairment and are commonly used as diagnostic indicators of renal dysfunction. The excessive secretion of albumin in the urine and its relation to the glomerulus is depicted in Fig. 1.

### Hyperglycemia

Another primary and independent risk factor for DKD is hyperglycemia [16]. The alteration in the antioxidant system and increased production of advanced glycation end products (AGE) results in continuous deterioration of renal capabilities. The pathophysiology of DKD is similarly thought to involve stimulation of the polyol pathway led by hyperglycemia flux and several linked microvascular problems [17]. Glycated hemoglobin (HbA1c) variation is associated with patients with diabetes (T1DM and T2DM). The onset and headway of diabetic nephropathy rely significantly on HbA1c [18]. An Italian multi-site trial, Renal Insufficiency and Cardiovascular Events (RIACE) reported a related finding [19]. In T2DM patients, forceful glucose control showed positive effects in postponing the development and stopping the progression of albuminuria [20, 21].

### Hypertension

A substantial risk factor for DKD is hypertension. A recent meta-analysis revealed that hypertension is ominously linked to the onset of diabetic nephropathy [21, 22]. Patients with hypertension are more likely to develop diabetic nephropathy, 95% confidence interval (CI, 13.1–2.14), than non-hypertensive patients [22]. A study from China based on a population perspective found that controlling hypertension can lower the risk of end-stage renal failure by 23%, further supporting this notion [23].

### Dyslipidemia

Dyslipidemia in diabetic patients is marked by a reduction in high-density lipoprotein and a rise in triglycerides and low-density lipoprotein [24]. Through the death of specialized epithelial cells (i.e., cells covering the surface of glomerular capillaries known as podocytes), clearance of dead adipocytes (macrophage infiltration), and increased extracellular matrix formation, dyslipidemia contributes to the progression of DKD [25]. The level of dyslipidemia may increase in DKD patients due to hyperglycemia and insulin resistance [26].

### Obesity

Obesity and diabetes-induced kidney disease (DKD) appear to be strongly correlated, according to the evidence [27]. However, the molecular mechanism of obesity causing DKD is not very well-defined; it is assumed that this risk factor causes proteinuria and glomerulonephritis (i.e., glomerular damage and renal hypertrophy) [28, 29].

### Smoking

It is characterized as a discrete key player to enhance the progression of DKD. Smoking plays a multifaceted pathogenic role in the expansion of DKD, which consists of oxidative stress, increased fats, cholesterol, triglycerides, protein glycation, accretion of AGEs, and protein loss into the urine (glomerulosclerosis) [30, 31]. It was discovered that smoking increased the possibility of diabetic nephropathy. This was further supported by a recent meta-analysis that found that T2DM patients who smoke have a higher chance of developing diabetic nephropathy. It was obtained by combining the data from nine group studies [32]. Furthermore, due to the close correlation between DKD and CVDs, patients with DKD have higher mortality rates [33]. Death and disease associated with DKD can have a significant impact on a person’s quality of life and increase the overall healthcare costs. Expenses of care for CKD patients are substantial, and these costs rise further in the manifestation of CVDs. Interventions that reduce or stop renal disease can benefit health care regimens targeting the condition [34]. Hence, it is important to appropriately treat comorbidities, including hypertension, dyslipidemia, and vascular dysfunction when managing DKD.

### Epidemiology

A substantial percentage of people with diabetes have DKD development. Since 2000, there has been an increase in the prevalence of diabetes; as of 2019, there were 463 million individuals with the disease worldwide, or 9.3% of people aged 20 to 79. By 2045, these figures are projected to be over 700 million and 10.9%, respectively [35]. Analyzing an average follow-up time of 24 years, the Diabetes Control and Complications Trial and the Epidemiology of Diabetes Interventions and Complications trial among T1DM subjects found that albuminuria developed in 23% of those receiving demanding treatment and 36% of those receiving conservative treatment [35]. The median line (15 years) of treatment and health condition update was done by the United Kingdom Prospective Diabetes Study. They found that 38% of T2DM subjects had developed albuminuria, and 28% had renal impairment [36]. According to regional research, the prevalence of CKD among people with diabetes ranges from less than 30% to beyond 80% [37]. Over the past 10 years, the prevalence of DKD in diabetic patients remained stable [38, 39]. However, the rising global trajectory of diabetes prevalence puts an increasing number of people in danger [40]. Globally, DKD is a significant contributor to end-stage renal disease (ESRD) in different countries, holding a record for 47% of new cases in the United States (US) and 60% in some nations like Malaysia and Singapore [41].

According to various cross-sectional studies conducted in the USA, there was no real revolution in the prevalence of DKD from 1988 to 1994 (28.4%) and 2009–2014 (26.2%). While the prevalence of albuminuria dramatically decreased in the USA throughout that time (from 20.8 to 15.9%), the rate of renal dysfunction described as a predictable eGFR < 60 ml min^−1^ 1.73 m^2^ notably rose from 9.2 to 14.1% [39]. The IDF calculated 8.4% of deaths with all causes in persons aged 20 to 79, or about 5.1 million, using statistics from the World Health Organization (WHO). A sensitivity analysis was conducted to adjust the relative risks of DM by 20%. The results of this analysis indicated that the estimate of DM-attributable mortality ranges from 5.1 to 10.1% of total mortality, with a range of 3.3 million to 6.6 million deaths globally. Additionally, the study also estimates that 1 in 12 global all-cause deaths is attributable to DM in adults [42].

## Pathophysiology of DKD

Great glycemic control is related to better kidney capability and more limited DKD advancement in diabetics [43]. Sustained hyperglycemia, or high blood sugar levels, can lead to a variety of detrimental effects on the kidneys. The formation of AGEs and oxidative injury can cause damage to the structure and function of the kidneys. Hypoxia, or lack of oxygen, can also occur as a result of poor blood flow to the kidneys. Metabolic and energetic disturbances can further contribute to kidney damage. Additionally, the overactivation of the renin-angiotensin-aldosterone system (RAAS) can cause hypertension and kidney damage. The production of inflammatory and fibrotic factors, such as transforming growth factor-beta (TGF-β), can also lead to the development of renal fibrosis, or scarring of the kidneys [44–46]. High blood glucose causes persistent metabolic and hemodynamic changes that modify signal transduction pathways, cytokines, chemokines, and growth factors. Furthermore, sustained hyperglycemia can lead to a number of negative effects on the body, including endothelial cell apoptosis. This is thought to occur through the activation of the nuclear factor kappa-light-chain-enhancer of activated B cells (NF-kB) and c-jun pathways. These pathways are involved in the regulation of inflammation and cell death, and their activation in response to hyperglycemia can lead to damage and dysfunction of the blood vessels in the kidneys. Understanding how metabolic pathways are altered in sustained hyperglycemia, or elevated blood sugar, is crucial for developing effective therapies for DKD [46].

In the early phases of DKD, persistent hyperglycemia can lead to a number of negative effects on the renal proximal tubule cells, which are responsible for reabsorbing glucose, amino acids, electrolytes, and other substances from the primary urine back into the bloodstream. High blood sugar levels can cause damage to these cells through various mechanisms, including the generation of reactive oxygen species (ROS), oxidative injury, and increased production of TGF-β. As a result of these demonstrations, all these effects lead to G1 cell cycle arrest of the proximal tubule cell and a senescent phenotype, which promotes interstitial inflammation and fibrosis [47, 48]. Vascular endothelial nitric oxide synthase (eNOS) aids in releasing the nitric oxide (NO). In the renal vasculature, NO is produced by the endothelial cells lining the blood vessels and acts as a vasodilator, meaning it relaxes the smooth muscle cells in the blood vessels and causes them to dilate. During DKD, a reduction in the synthesis of nitric oxide happens. This leads to lower levels of NO in the blood vessels of the kidneys, making them more sensitive to vasoconstriction. This vasoconstriction is caused by other factors such as the peptide hormone endothelin-1, which can further contribute to the progression of DKD [49].

**Fig. 2:**
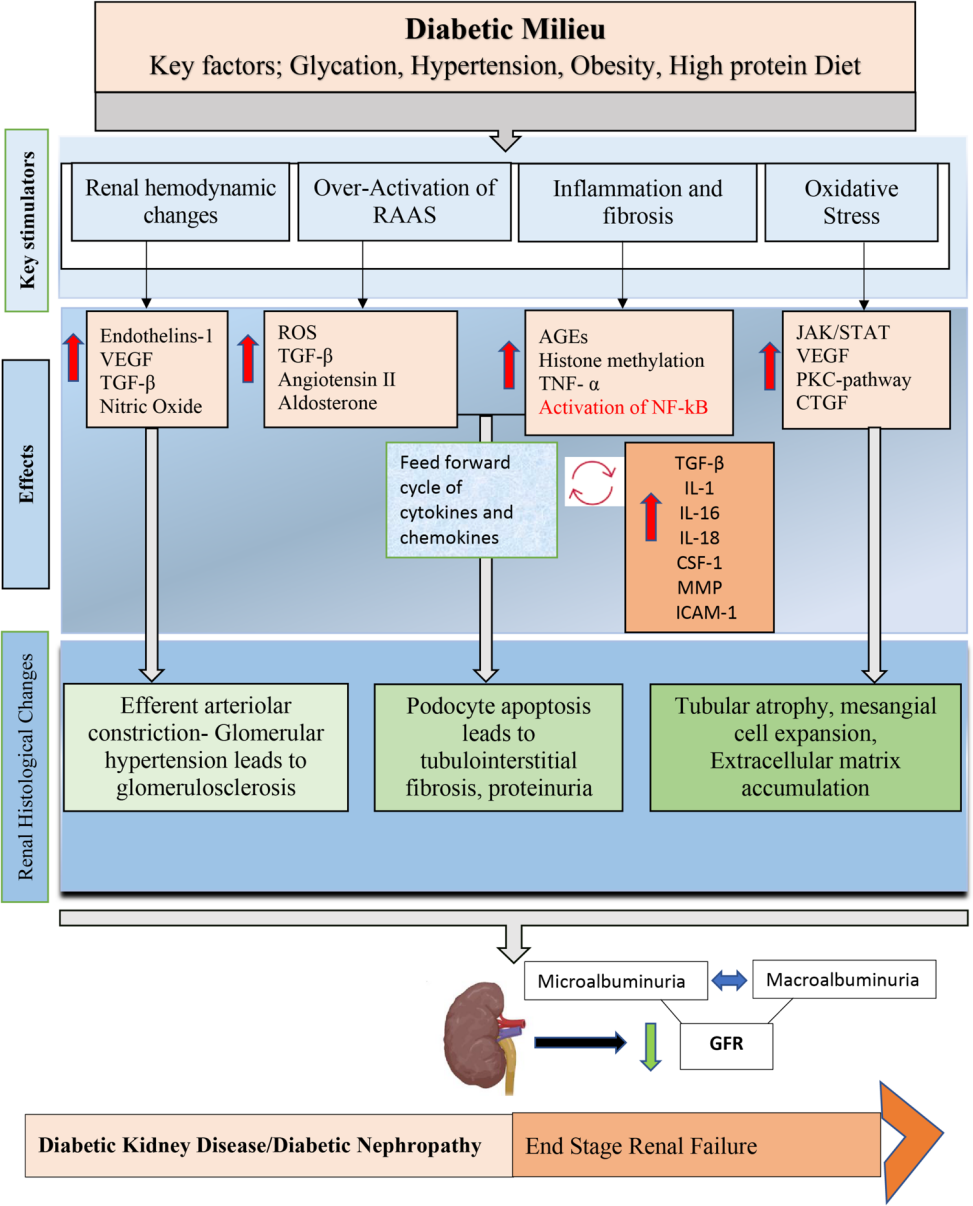
Diabetic kidney disease is characterized by metabolic and hemodynamic aberrations, which interact between several ROS-related pathways. In diabetes, the regulation and activation of particular growth or transcription factors are influenced by metabolic abnormalities, hemodynamic mediators, and the creation of ROS. The magnitudes of the activation or inhibition at the molecular or metabolic level give rise to the histological (structural and functional) changes causing diabetic kidney disease. IL, interleukins; ECM, extracellular matrix; TGF, transforming growth factor; AGEs, advanced glycation end products; GFR, glomerulus filtration rate; CTGF, connective tissue growth factor; MMP, matrix metalloproteinases; ROS, reactive oxygen species

Aberrations such as mitochondrial dysfunction, apoptosis, breakdown of autophagy, and activation of intracellular signal transduction pathways, such as protein kinase C (PKC) and mitogen-associated protein kinase (MAPK), which also interact with NF-κB in the renal tubules, have been observed. This incorporates support of inflammatory cytokines and chemokines (interleukins: IL-1, 6, 18), tumor necrosis factor (TNF-α), monocyte chemoattractant protein (MCP)-1, macrophage colony-stimulating factor (CSF-1), and macrophage inflammatory factor (MIF). Large-scale manufacturing of cytokines that encourage fibrosis, like TGF-β and CTGF (connective tissue growth factor), has additionally been connected to DKD [50]. The concise pathophysiology of DKD is illustrated in Fig. 2. Diabetes-related hemodynamic changes may increase the possibility of acute kidney damage in DKD-sick people [51]. Many studies have shown that insulin resistance is a different potential risk for acute kidney injury. As a result, it is critical to monitor the beginnings of renal damage in patients with diabetes, as this disorder can quicken the progression of CKD. DKD is also caused by a crucial metabolic transformation in the body, which causes damage to body parts other than the kidney, e.g., the liver, skeletal system, arteries, nerves, and body fat. The damage of these organs can lead to the list of comorbidities which are associated with diabetes. The significance of epigenetic modifications in the early phases of DKD has been underlined by the latest researches, which may impact transcriptional activity from different pathways, thereby modifying the disease [52].

## Treatment

DKD is a common complication of diabetes and can lead to abnormal levels of blood pressure, lipids, and glucose, which can further damage the kidneys and increase the risk of cardiovascular disease. It is important for individuals with diabetes to closely monitor and manage their blood sugar levels and to work with their healthcare provider to manage any other risk factors [53–57]. A lifestyle change is crucial for the diagnosis, control, and therapy of DM and related consequences [58].

### Controlling level of blood glucose

It is estimated that about 20% of people with DM developed DKD irrespective of well-controlled blood glucose levels [59]. Certain clinical studies have shown that intensive blood glucose control can delay the progression of diabetic kidney disease and protect renal function such as development of microalbuminuria and reduced eGFR in diabetic patients [60]. To gentle the progression of DKD, initial DM patients must toughen glycemic control to eliminate the quantity of HbA1c to 7.0% or less, according to regulations from the American Diabetes Association (ADA)/European Association for the Study of Diabetes (EASD) [61] and Kidney Disease Outcomes Quality Initiative (KDOQI) [60]. However, some studies have found that HbA1c levels below 6.0% or above 9.0% are associated with an increased risk of death [62, 63]. Therefore, current international guidelines recommend an individualized approach to treatment intensity based on the patient’s characteristics and risk factors. This means that while the target HbA1c of 7% is a general recommendation, the individualized approach to treatment intensity will be taken into consideration which means the target may vary for different patients [60].

The significance of blood glucose control cannot be overstated. It directly influences the effectiveness of various therapeutic approaches, including stem cell therapies such as MSC-based treatments for diabetes. An illustrative example of this is highlighted in the meta-analysis conducted in 2016 [64]. Their study underscores the critical role of glucose control and its direct impact on the outcomes of stem cell therapy for diabetes. Notably, the study raises a pivotal consideration: the potential challenges associated with MSC-based treatment in the presence of diabetic ketoacidosis (DKA) if not managed correctly. The data from their meta-analysis suggests that individuals with DKA at the time of diagnosis may present unique challenges for stem cell therapy. One possible explanation for this suboptimal clinical response among DKA patients is the presence of very low β-cell reserve. Therefore, the success of MSC-based treatments for diabetes is intricately linked to meticulous glucose control and the avoidance of complications associated with DKA. These findings reiterate the importance of precise glucose management and patient selection within the context of stem cell therapy for diabetes, reinforcing the significance of individualized care.

### Controlling body weight

Excessive body weight leads to insulin resistance, systemic inflammation, and metabolic dysregulation which in turn establish the detrimental effects on renal function. Thus, successful weight management, attained through lifestyle adjustments, dietary regulation, and routine physical exercise, serves as a crucial means not only to enhance glycemic control but also to diminish the risks and progression of DKD. As we delve into the multifaceted approach of DKD management, it is important to consider the innovative approaches such as the therapeutic potential of mesenchymal stem cells (MSCs) in the context of obesity, diabetes, and its related complications. In numerous investigations, the administration of human MSCs or MSC-derived conditioned media to diet-induced obese (DIO) mice resulted in a notable reduction in both body weight and fat mass. What is more, MSC therapy demonstrated a significant improvement in the metabolic parameters of obese mice, including enhanced insulin sensitivity and reduced levels of blood glucose and triglycerides [65]. Consequently, multiple MSC administrations shielded obese mice from the onset of metabolic syndromes linked to obesity, including diabetes, fatty liver disease, and cardiovascular impairment [66]. In another study, intravenous administration of human adipose-derived MSCs was employed in high-fat-diet-induced obese mice. This intervention led to reduced adipose tissue weight, adipocyte size, and fat mass, along with improved metabolic profiles. Additionally, it increased energy expenditure, upregulated metabolic genes, and induced a shift towards anti-inflammatory M2 macrophages in adipose tissue [67].

### Blood pressure control

Strict blood pressure supervision in DKD patients can substantially lower albuminuria, delay loss of kidney function, and minimize the risks of heart disease [68]. The American Diabetes Association (ADA) recommends that people with diabetes aim to keep their blood pressure under 140/90 mmHg (1 mmHg = 0.133 kPa). For patients with cardiovascular disease or kidney failure, a lower blood pressure target of 130/80 mmHg is recommended. All other patients control their blood pressure at a target of 130/80 mmHg [69]. The Action to Control Cardiovascular Risk in Diabetes (ACCORD) blood pressure trial was a large randomized controlled trial that recruited participants with type 2 diabetes and randomized them to an intensive blood pressure therapy group or a standard blood pressure therapy group. The intensive therapy group aimed to achieve a systolic blood pressure of less than 120 mmHg, while the standard therapy group aimed to achieve a systolic blood pressure of less than 140 mmHg. The trial found that intensive blood pressure therapy reduced the incidence of cardiovascular events and overall mortality in the intensive therapy group compared to the standard therapy group. These findings support the use of more aggressive blood pressure targets in patients with type 2 diabetes to reduce the risk of cardiovascular disease [70–72].

Angiotensin-converting enzyme inhibitors (ACEIs) and angiotensin II receptor blockers (ARBs) are commonly used as first-choice remedies for the treatment of DKD due to their ability to slow the progression of kidney damage and reduce the risk of cardiovascular disease. These drugs work by blocking the action of the angiotensin II hormone, which plays a key role in the development of kidney damage and hypertension. However, it is important to note that the combination of an ACEI with an ARB is generally contraindicated as it has been shown to increase the risk of kidney injury and hyperkalemia [73–75]. It has been reported that dual blockade of RAAS by using a combination of an ACEI and an ARB can lead to significant drops in blood pressure and severe renal failure in animal models, such as in spontaneously hypertensive rats receiving a low-sodium diet. The RAAS is a complex hormonal system that plays a key role in regulating blood pressure, electrolyte balance, and fluid volume in the body. Blocking the RAAS with drugs like ACEIs and ARBs can lead to decreased blood pressure and improved kidney function, but when both pathways are blocked, it can lead to a decrease of blood pressure to dangerous levels and severe renal failure [76].

According to the review outcomes of the ALTITUDE (Aliskiren Trial in Type 2 Diabetes That using Cardio-Renal Endpoints NCT00549757), these appointed subjects randomly in a controlled trial and evaluated the use of aliskiren as adjunctive therapy to an ACEI or ARB in individuals with T2DM, CKD, or CVDs. This trial elucidated that aliskiren as an alternative treatment regimen did not further enhance renal performances; instead, it slowed the succession of albuminuria [77, 78]. Unfortunately, the trial was terminated early due to an increased risk of adverse events in the aliskiren group compared to the placebo group. Compared to existing treatments, ACEI and ARB combined extended the major unfavorable responses, i.e., extreme kidney injury and hyperkalemia, in a comparable study called VA-Nephron-D (Veterans Affairs Nephropathy in Diabetes, NCT00555217). From the start of the therapy to the 2-year follow-up, the danger of serious kidney harm was likewise immeasurably greater [74, 79]. When taking medication for ACEI/ARB, the urine albumin to creatinine ratio (UACR), creatinine clearance (CCr), potassium levels in the blood, and serum amount must all be surveilled.

### Control of blood lipids

DKD patients are highly compromised with vascular problems, which may lead to hyperlipidemia. It can disrupt the barrier function of endothelial cells while also endangering blood supply [80, 81]. The KDOQI guidelines advise statin-based treatment for lowering low-density lipoprotein cholesterol (LDL-C) levels to lessen the risk of atherosclerotic complications in DKD patients. According to a meta-analysis of cholesterol treatment, the incidence of significant cardiovascular events decreased by 23% for each mmol/L drop in LDL-C [82]. The “2019 ESC Guidelines on diabetes, pre-diabetes, and cardiovascular diseases development in collaboration with the EASD” recommend that in patients with arteriosclerotic cardiovascular diseases and diabetes or stage 3–4 CKD, LDL-C should be controlled to a target of less than 1.4 mmol/L (or 55 mg/dL) to reduce the risk of cardiovascular disease. This is a stricter target compared to the general population where the target is less than 2.5 mmol/L [83].

### Low-protein diet

The reductions in proteinuria (the amount of protein in the urine) have been shown to be a comprehensive index of decreased renal and cardiovascular event risk in T2DM patients [84]. Regardless, a low-protein eating plan for DKD has ignited some talk. The American Diabetes Association (ADA) recommends that people with DKD should aim to control their protein intake by consuming about 0.8 g/kg of body weight per day (0.8 g kg^−1^ d^−1^). This is in contrast to higher levels of protein intake, which have been found to accelerate the decline of kidney function as measured by the glomerular filtration rate. This is because higher levels of protein intake can cause an increase in metabolic waste products that the kidneys must filter, which can lead to further damage [85]. Ingesting less protein, then again, would gainfully affect glucose, GFR power, or cardiovascular issues [86].

### Use of new hypoglycemic drugs

RAAS (ACE inhibitors and ARBs) and metformin (anti-diabetic medication to control the glucose levels) can help to reduce the workload on the kidneys. These medications may be used together or as monotherapy to help slow the progression of DKD and protect kidney function [87, 88]. The development of DKD is undeniable and may require an urgent need for a novel treatment approach [89]. The pathological and molecular mechanisms of DKD are under study to develop more target based-drugs [90–93]. Three novel hypoglycemic remedies, including dipeptidyl peptidase-4 (DPP-4) inhibitors, sodium-glucose cotransporter-2 inhibitors (SGLT2i) receptor agonists, and glucagon-like peptide 1 receptor agonists (GLP1-RA), show comprehensive renal protective effects [94]. Clinical trials have shown promising results, particularly for hypoglycemic drugs/inhibitors, such as SGLT2 inhibitors, GLP-1 agonists, and DPP-4 inhibitors. In an animal or human clinical assessment, solutions like protein kinase C inhibitors, AGE inhibitors, endothelin receptors, Rho kinase, and TGF-β have shown promising positive effects and bring hope for the treatment of DKD. Other novel medicines that are still being researched include CCX140b [94], PF-00489791 [95], and NOX-E36 [96]. Moreover, current preclinical studies are investigating the patterns of multiple novel targets, including adiponectin and its receptors [97], NADPH oxidase [98], histone deacetylase [99, 100], and microRNA [101, 102] in DKD.

Currently, most drugs are being tested at different degrees of advancement. To predict DKD patients, there is a dire need to conduct extensive studies that utilize larger cohorts and randomly controlled trials. The pathophysiology of DKD is not yet fully comprehended, and there are only a few treatments that target signaling molecules or pathways. Moreover, while certain drugs have demonstrated efficacy in preclinical studies, they either do not advance to clinical trials or lack sufficient clinical trials to establish their effectiveness. Despite some drugs showing promising results for DKD, their safety profiles remain insufficient. As a result, we have a long way to go in treating DKD given these challenges. Traditional methods for treating DKD, like conventionally prescribed medicines, physiotherapy, and dietary treatment, are not always fruitful. To lessen the significant patient count and medical services load, innovative therapy models like cellular therapy are required. These techniques may hamper the development of DKD and contribute to repairing the defected organs without causing any severe side effects [74]. Mesenchymal stem cells (MSCs), i.e., adult stem cell treatment, are possibly the most favorable cellular therapy in the field of regenerative medicine. As they may be used systemically or locally to treat many diseases, due to their self-renewal and differentiation potential.

### Mesenchymal stem cell therapy

The capacity to self-renew and give rise to cells from different lineages are two good qualities of stem cells. Interaction of MSCs with numerous human illnesses like diabetes mellitus, malignant growth (cancer), and neurodegenerative diseases like Parkinson’s and Alzheimer’s has been widely investigated. Stem cells can be classified based on their origin; embryonic stem cells, adult stem cells, and induced pluripotent stem cells. Adult stem cells are undifferentiated cells found in various tissues in the body that have the capacity to differentiate into multiple cell types of that specific tissue or organ. These stem cells incorporate MSCs, HSCs (hematopoietic stem cells), satellite cells, and MuSCs (muscle stem cells). Traditionally, MSCs were first isolated from bone marrow (BM-MSCs) and spleen from guinea pigs [103].

MSCs may generally be observed in the bone marrow, birth canal, umbilical cord blood, visceral fat, brain tissues, and several other tissues. Research has highlighted that MSCs are highly variable, exhibiting significant heterogeneity among various sources and even within the same source, which underscores the need for thorough characterization and quality control measures. Considering this, the International Society for Cellular Therapy (ISCT) made specific standards for all MSCs separated by various sources. In 2019, the ISCT introduced updated guidelines that aim to address the inherent heterogeneity of mesenchymal stem cells (MSCs). These guidelines encompass a variety of analytical methods designed to illustrate various functional properties of MSCs. These properties include the secretion of trophic factors, the modulation of immune cells, and their role in angiogenesis. The ISCT MSC committee has put forth recommendations for research studies in this field. These recommendations emphasize the importance of providing comprehensive information in the following key areas (i) tissue source origin (to highlight the tissue-specific properties associated with these cells), (ii) stemness properties (described by both in vitro and in vivo data), (iii) functional assays (employ a robust set of functional assays to assess the properties of MSCs in relation to their intended therapeutic mechanisms). Furthermore, evaluating MSC-based products involves a set of fundamental assays, which encompass donor screening; viability assessments; purity tests (including assessments for residual contaminants and pyrogenic/endotoxin presence); safety evaluations (such as bacterial, fungal, mycoplasma, viral tests, and tumorigenicity assays); identity assessments (including immunophenotypic profiles); and potency tests (comprising evaluations of multilineage differentiation, secretion profiles, CFU-f assays, and immunosuppressive properties). Incorporating these ISCT recommendations is crucial to ensuring that research in this field adheres to established standards and guidelines for the characterization and assessment of MSCs. These guidelines serve as a foundational reference point for evaluating the quality and therapeutic potential of MSC-based interventions across various clinical applications [104]. As per the ISCT’s MSC criteria, genuine human MSCs should not display specific surface markers, which include HLA-DR (MHC class II), CD19, CD14, CD34, CD45, and the B-cell antigen receptor’s alpha chain, CD79a. Conversely, MSCs are expected to exhibit the presence of positive surface markers such as Thy-1 (CD90), 5′-nucleotidase (CD73), and endoglin (CD105). In an in vitro setting, MSCs should also showcase their ability to undergo differentiation into adipogenic, osteogenic, and chondrogenic lineages while manifesting the corresponding phenotypic characteristics [105]. Notwithstanding these attributes, MSCs additionally produce bioactive substances with immunoregulatory properties that advance tissue redesigning and fixing.

### MSCs origin and expansion

The most common isolation sources of MSCs include adipose tissue, bone marrow, and umbilical cord. Certain in vitro and in vivo studies subjected their investigation routes on the regenerative potential of MSCs derived from aged and young donors. The majority of the investigations looked at how donor age correlated with MSC performance, which can range from 16 months to 90 years old. The studies showed that samples taken from older individuals exhibit lower MSC frequency, colony forming unit (CFU) efficiency, population doubling rate, osteogenic, and differentiation potential with increased risks of senescence. Senescence in MSCs can greatly impact their regenerative potential, reducing the ability to differentiate into specific cell types, to recruit macrophages, and to polarize them towards the anti-inflammatory M2 phenotype. This highlights the importance of obtaining MSCs from young and healthy donors when cultivating them for therapeutic purposes, and to avoid the expansion of senescent cells [106].

### Umbilical cord MSCs

In the recent decade, MSCs derived from umbilical cord (UC) blood, placenta, and amnion have presented numerous advantages over MSCs acquired from bone marrow and adipose tissues (AT). A higher proliferation rate, increased proliferation capacity, and longer lifespan in comparison to BM and AT-MSCs was observed. Previously conducted studies have also observed the expansion capacities of UC-MSCs as they can be proliferated over several passages (up to 16) with the retention of normal karyotype and avoiding the senescence [107, 108]. Conversely, there are some shortcomings to successfully isolating and expanding UCB-MSCs; most notably the low frequency of MSC clones compared to UC, which has a rich supply of highly proliferative MSCs that are characterized by: compared to BM-MSCs, it has a uniform phenotype of adherent, spindle-shaped fibroblast-like cells in primary culture, a higher isolation yield (5 × 10^4^–5 × 10^5^ cells from 1 cm^3^ of UC tissue), a higher frequency of colony forming units (CFU)-F, and a shorter DT (24 h) [109].

Furthermore, the UC-MSCs secretome maybe comparable to the BM-MSCs secretome in its abundance of angiogenic factors. However, a previously conducted study points out that the isolation of MSCs, cell freezing conditions, and exposure of cells in cryoprotectants still needs further research [110]. Future research stating the pros and cons of using fetal tissue-derived MSCs requires large-scale investigations. Similarly, another study presented their findings regarding MSCs derived from young donors. They observed a faster proliferation rate, shorter doubling time, and efficiency in secreting cytokines for tissue regeneration in comparison to MSCs obtained from older or adult donors. The MSCs obtained from aged individuals presented increased chromosomal instability, employing genetic changes or mutations. Also, they were more susceptible to oxidative stress, which can lead to damage to DNA, proteins, and other cellular components and can negatively impact the cells’ ability to function properly. The idea that aging affects cell proliferation holds great importance. Cell division and proliferation capacity slows down as we age due to morphological and cellular changes. The presence of aging-related markers like SA-β-gal, P16, and p21 may also be the cause of the observed differences in proliferation between MSCs of varying ages [111]. It is important to remember that MSCs are a type of adult stem cell that can grow into many different kinds of cells, but where they origin from, affects the therapeutic purpose.

### iPSC-MSCs

The indefinite in vitro culture capability of induced pluripotent stem cells (iPSCs) and their potential to differentiate into various cell types, including MSCs, make them a promising alternative to BM-MSCs for cell and gene therapy applications. The study [112] showed that iPSC-MSCs can be generated in clonal expansion and differentiated into various cell types including osteoblasts, adipocytes, and chondrocytes, and promote tissue regeneration. This enhanced regenerative potential and decreased senescence of iPSC-MSCs has been suggested to be due to higher telomerase activity compared to BM-MSCs, which may contribute to a decrease in the possibility of losing potency in the long-term culture of MSCs. Additionally, the iPSC-MSCs have the advantage of being derived from autologous cells which makes them more suitable for therapies in personalized medicine. Also, future clinical trials would better unveil the actual results of this domain [113].

### Molecular mechanisms of MSC-based therapy for DKD

Experimental studies have shown promising results in using MSCs for relieving DKD, as outlined in Table 1, although the exact molecular mechanisms are still being investigated. MSCs are multipotent cells that can differentiate into various cell types, including glomerular endothelial cells, when stimulated appropriately. The process of homing MSCs to damaged kidneys involves several molecules, including chemokine receptors, adhesion proteins, and the matrix metalloproteinases (MMPs) family, with stromal cell-derived factor-1 (SDF-1) and its receptor CXCR4 playing a significant role in MSC migration to the site of kidney damage [114]*.* In vitro studies have found that MSCs display a unique cellular behavior known as nonapoptotic membrane blebbing, which is similar to that of metastatic tumor cells. This behavior allows MSCs to migrate through the endothelium and overcome the basal barrier through the action of MMPs, particularly MMP2 and MT1-MMP. This allows MSCs to move through the tissue barriers and reach the damaged site [115]. However, despite the potential of MSCs to migrate to injured tissue and differentiate into functional replacement tissue, most studies have shown that only a small fraction of systemically administered cells can actually achieve this. Moreover, only a small percentage of transplanted cells can successfully differentiate into functional tissue. Additionally, the administered cells are almost undetectable in other organs within 24 h, suggesting that their therapeutic effects may be mainly due to their paracrine activity rather than their differentiation potential [116]. Therefore, more research is needed to fully understand the molecular mechanisms underlying MSC-based therapy for DN and to improve the efficacy of this approach.

**Table 1:** MSCs administration for DKD in preclinical studies

**MSCs type**	**Model**	**DM induction features**			**DM/DKD criteria**	**Obesity settings**	**Study groups (*n*)**	**Frequency and dose of MSCs**	**Injection method**	**Change in body weight**	**Outcomes**	**Ref**
		***Dose***	***Route***	***Type***								
**PMSC**	Male SD rats	Single-STZ (60 mg/kg) injection	IP	T1DM	If blood glucose ≥ 16.7 mmol/L (72 h after injection). DKD was conformed after the 8th week of STZ injection	Adaptive feeding for 1 week	Control (10) + DKD-MSC (10)	1 × 10^6^ cells Once a week for 3 weeks	Tail vein injection	N/A	PMSCs showed reduced SCr, BUN, cystatin C levels, and UACR. Alterations in mesangial matrix expansion, filtration barrier injury, and tubular vacuolar degeneration. They modulated the Th17/Treg balance, reducing pro-inflammatory cytokines, and exhibited selective recruitment to immune organs. In vitro, PMSCs rebalanced the Th17/Treg ratio by upregulating PD-1 and downregulating PD-L1.	[121]
**PMSC**	Male SD rats	Singe-STZ (60 mg/kg) injection	IP	T1DM	If RBG > 16.7 mmol/L (72 h after injection). DKD was conformed after the 8th week of STZ injection	N/A	DKD (10) + PMSCs (10)	1 × 10^6^ cells	Tail vein injection	N/A	PMSCs can help reduce podocyte injury in DKD. They have a therapeutic role by enhancing mitophagy and SIRT1-PGC-1α-TFAM signaling in podocytes, i.e., regulating mitochondrial homeostasis.	[122]
**AMSC**	Male SD rats	Single-STZ (55 mg/kg) injection	IP	T1DRM	If BGL ≥ 16.65 mmol/L; 24 h urine protein ≥ 30 mg (72 h after STZ injection)	Standard diet	Normal (24) + DKD (24) + PBS-hAMSCs (24)	2.0 × 10^6^ cells	Penile vein	Decreased in the MSC group	Transplanted hAMSCs homed to injured kidneys, increasing ARFs and lowering blood glucose. They improved kidney function, reducing urinary protein (24 h), SCr, urea, and KIM-1 while alleviating renal pathology.	[123]
**UCMSC**	Male BALB/c mice	Single-STZ (150 mg/kg) injection	IP	T1DM	If glucose conc. > = 16.7 mM (48 h after STZ injection)	Standard diet	Normal (6) + DM (8) + DM-MSC (8)	1.0 × 10^4^/g/kg weekly for 4 weeks	Tail vein injection	Increased	Repeated injection with mUC-MSCs was found to alleviate albuminuria, glomerulus injury, and fibrosis in DN mouse models.	[124]
**UCMSC**	Male SD rats	Single-STZ (60 mg/kg) injection	IP	T1DM	If RBG level > 16.7 mmol/L (6 weeks after STZ injection)	Adaptive feeding for 1 week	Control (5) + DKD (5) + DKD-UCMSC (5)	2 × 10^6^ cells once a week for twice	Tail vein injection	Decreased in the MSC group	UC-MSCs were found to inhibit 24-h urinary total protein, urinary albumin to creatinine ratio, serum creatinine, and blood urea nitrogen, as well as attenuate pathological abnormalities.	[125]
**UCMSC**	Male CD1 mice	Single-STZ (80 mg/kg) injection	IP	T1DM	If increased UACR from the threshold after the 5th week of STZ injection	N/A	Control (3) + DM (3) + DKD-MSC (3)	5 × 10^6^ Three doses (5, 9, and 13 weeks after STZ)	Tail vein injection	N/A	UC-MSCs can prevent the progression of DKD by addressing mitochondrial dysfunction in TECs and induce the production of a protein called *Arg-1* in macrophages, which leads to the reversal of mitochondrial dysfunction in TECs. **↓** Renal mRNA desmin, αSMA, Fn1, Kim-1, NGAL, MCP-1, VCAM-1, ICAM-1, IL-1b, TNF-α, IL-6, and iNOS	[126]
**UCMSC**	Rhesus macaques	Single-high dose STZ (80 mg/kg) injection	IV	T1DM	If FBG levels at 15–20 mmol/L	HFD + HSD (2 years)	Control (3) + DKD (4) + DKD-MSC (8)	2 × 10^6^ cells/kg (4 doses; 2-weeks apart)	IV injection	N/A	MSCs influence the expression of a protein called SGLT2, which is involved in glucose transport in the kidneys. By influencing this expression, the MSCs are able to improve glycemic control. MSC treatment exhibited lower fasting blood glucose, HbA1c levels, and insulin requirements, as well as decreased SCr and BUN levels, though the protective effects waned after one course of treatment.	[127]
**UCMSC**	Male SD rats	Single-STZ (60 mg/kg) injection	IP	T1DM	If BGL ≥ 16.7 mmol/L for 3 days; 24 h urine protein ≥ 30 mg/kg for DKD after 6th week of STZ injection	Adaptive feeding	Control (5) control + UCMSC (5) + DKD (5) + DKD-UCMSC (5)	2 × 10^6^ cells for twice	Tail vein injection	N/A	UC-MSCs appeared to reduce kidney damage and inflammation. They aided in suppressing pyroptosis in the renal tubular epithelial cells by downregulating miR-342-3p, a regulator of the NLRP3/caspase1 signaling pathway.	[128]
**UCMSC**	Male SD rats	Single-STZ (50 mg/kg) injection	IP	T1DM	If BGL conc. ≥ 16.1 mM for 3 days after 1 week of STZ injection	Standard feeding conditions	Normal (8) + DM (8) + MSC-L (8) + MSC-H (8)	5 × 10^6^ cells; 1 × 10^7^ cells	IV injection	Increased in MSC groups	The sustained decrease in body weight was seen after hucMSCs. It has also exhibited distribution of hucMSCs within the kidneys, conferring substantial protection in the in vivo and in vitro context; these cells enhanced cell viability and wound healing and mitigated senescence in rat podocytes damaged by high glucose. These effects were attributed to paracrine action, implicating the activation of the AMPK/mTOR pathway	[129]
**BM-MSC**	Female Wistar rats	Single-STZ (60 mg/kg) injection after overnight fasting	IP	T1DM	If RBG after 72 h is > 16.7 mmol/l for 3 days after 8th-week of STZ injection	Adaptive feeding	DKD (4) + MSC (4) + MSC-ACE2 (4)	2 × 10^6^ Once a week for 2 weeks	Tail vein injection	N/A	Modifying MSCs using ACE2 has been shown to have the potential to reduce glomerular fibrosis and inhibit renal RAS activation.	[130]
**BM-MSC**	Male SD rats	Single-STZ (55 mg/kg) injection	IP	T1DM	If FBG levels > 11.1 mmol/L for 2 days	Regular diet (10% fat, 20% protein, and 70% carbohydrate)	Control (7) + DKD-saline (7) + DKD-MSC (7)	1 × 10^7^ cells/kg per week for 6 weeks	Tail vein injection	Increased in MSC group	The protective effect of MSCs on the kidneys of DN rats may be related to CD8(+) T cell immunosuppression mediated by CD103(+) DCs.	[131]
**BM-MSC**	Male BALB/c mice	Single-STZ (150 mg/kg) injection	IP	T1DM	If blood glucose level ≥ 16.7 mmol/L	N/A	Control (6) + DKD (6) + DKD-MSC (6)	5 × 10^5^ cells once for 2 weeks	Tail vein injection	Increased	MSCs can reduce inflammation and improve kidney injuries in DN mice through the activation of a protein called transcription factor (TFEB). This activation leads to a switch from M1 macrophages (which promote inflammation) to M2 macrophages (which are involved in tissue repair). The end result is a decrease in inflammation and an improvement in renal injuries.	[132]
**BM-MSC**	Male SD rats and male C57BL/6 mice	STZ (150 mg/kg) injection in rats; STZ (55 mg/kg) in mice	IP, tail vein	T1DM	1 week after STZ injection if BGL ≥ 400 mg/dl for STZ-diabetic mice, or ≥ 300 mg/dl for STZ-diabetic rats	Adaptive feeding	Control (3) + MtDsRed2-MSCs (3) + DsRed2-Mt (3)	1 × 10^4^/g/body weight every 2 weeks in STZ-mice; 2 × 10^6^ cells in STZ-rats	IV, under renal capsule injection	N/A	BM-MSCs transfer their mitochondria to damaged proximal tubular epithelial cells, which may help to restore cellular function and reduce further damage.	[133]
**BM-MSC**	Male albino Wistar rats	Single-STZ (50 mg/kg) injection	IP	T1DM	DKD indication was done if 24 h UAE > 30 mg/day at the 6th week after STZ injection	Standard rat chow diet	Control (10) + DKD (10) + DKD-MSC (10) + DKD-MSC-MT (10)	1 × 10^6^ labeled MSCs	Tail vein injection	N/A	Pretreatment with melatonin enhances the proliferation and efficiency of the BM-MSCs, which may contribute to their therapeutic effects. By improving kidney function, the BM-MSCs pretreated with melatonin may be able to ameliorate the symptoms of DKD in the rat model.	[134]
**BM-MSC**	Male SD rats	Single-STZ (55 mg/kg) injection after 12 h fasting	IP	T1DM	After 72 h of STZ injection, If RBG > 16.7 mmol/L	Adaptive feeding for 1 week	Control (10) + DKD (10) + DKD-MSC (10)	2 × 10^6^/mL after 4 weeks of DM	Tail vein injection	Decreased in the MSC group	BM-MSCs were shown to reduce the levels of PAI-1 protein, which is involved in the progression of fibrosis, and decrease the accumulation of ECM (extracellular matrix) in the kidney	[135]
**BM-MSC**	Male tree shrews	STZ (100 mg/kg) injection after 12 h fasting	IP	T2DM	DKD was detected if FBG ≥ 11.1 mmol/L for 12 weeks	HFD and high-sugar diet	Control (10) + DKD (6) + DKD-MSC (6)	5 × 10^6^/kg labeled cells injection for twice	IV injection	Decreased	Transplantation of MSCs was found to improve kidney and pancreas function by homing to the injured organs. This resulted in a reduction of 24-h proteinuria and an improvement of insulin resistance.	[136]
**BM-MSC**	Female Wistar rats	Single-STZ (60 mg/kg) injection after 24 h fasting	IP	T1DM	If RBG after 72 h is > 16.7 mmol/l at 3 continuous days after 8 weeks of STZ injection	Adaptive feeding	Control (16) + DKD (16) + DKD-MSC (16)	Two doses: 2 × 1 0^6^ (1 week apart)	Tail vein injection	Decreased in the MSC group	MSC treatment reduces ROS generation by lowering blood glucose levels, suppressing the expression of TGF-β and cellular glucose uptake through GLUT1, consequently inhibiting oxidative stress in diabetic kidneys.	[137]
**BM-MSC**	Male SD rats	Single-STZ (65 mg/kg) injection after overnight fasting	IP	T1DM	If BGL > 16.7 mmol/L. Diabetic rats received insulin (1–4 U/rat) injections daily to maintain BGL (16–28 mmol/L) and avoid ketonuria	Adaptive feeding for 1 week	Control (6) + DKD (10) + DKD-MSC (14)	Single dose, 2 × 10^6^	Left renal artery injection	Unchanged	MSCs have been shown to attenuate podocyte injury and resulted in significant alterations, such as reduced kidney weight and kidney-to-body weight ratio, elevated creatinine clearance, reduced albuminuria, enhanced renal histology, and increased levels of renal nephrin, podocin, VEGF, and BMP-7.	[138]
**AD-MSC**	Male SDT fatty rats			T2DM	Unilateral nephrectomy to accelerate DKD progression in SDT fatty rats	-	Control (6) + DKD-MSC (7) + DKD-ASC sheets (8)	6 × 10^6^/ml Single dose	IV and cell sheets directly into the kidney	N/A	Engraftment of AD-MSC sheets directly into the kidney was found to improve transplantation efficiency and inhibit the progression of renal injury.	[139]
**AD-MSC**	Male SD Rats	STZ (40 mg/kg) injection for 5 days	IP	T1DM	2 weeks after STZ injection, if glucose concentrations > 300 mg/dl	Standard diet	Control (8) + DKD (8) + DKD-MSC (8) + vehicle (8)	1 × 10^7^ cells	Tail vein injection	N/A	Engraftment of AD-MSCs may alleviate renal injury in DKD by activating a protein called klotho and inhibiting the Wnt/β-catenin pathway. This protein that has been shown to have protective effects on the kidney, and its activation by the AD-MSCs may contribute to the alleviation of renal injury. The Wnt/β-catenin pathway is a signaling pathway that has been implicated in the development of DN, and its inhibition by the AD-MSCs may further contribute to their therapeutic effects	[140]
**AD-MSC**	Male SD rats	CKD via nephrectomy and arterial ligation of 2/3rd of left kidney followed by STZ (30 mg/kg) and aminoguanidine (180 mg/kg) injections	IP	T2DM	1 week after STZ injection if blood glucose level ≥ 250 mg/dL	Adaptive feeding	SC (6) + DKD (6) + DKD-EMPA (6) + DKD-ADMS (6) + DKD-AD-MSC-EMPA (6)	DKD-EMPA/20 mg/kg/day at 14th day of CKD-induction, DKD-AD-MSCs 6.0 × 10^5^ at 28th day, followed by 1.2 × 10^6^ at 35th and 42nd day after CKD-induction	Intrarenal arterial and IV injections	N/A	Combination therapy involving AD-MSCs and the EMPA group demonstrated superiority over individual treatments (Sham, DKD, DKD + EMPA group) in safeguarding kidney function and preserving ultrastructural integrity in DKD rodents. The combined treatment led to notable enhancements in blood glucose levels, circulatory BUN/creatinine ratios, urine protein/creatinine ratios, and several biomarkers linked to inflammation, oxidative stress, apoptosis, fibrosis, and mitochondrial damage.	[141]
**SHED**	SPF-Grade 12 GK male rats			T2DM (non-obese model)	If non-fasting BGL ≥ 11.1 mM for 3 consecutive days were classified as DKD rats	HFD for 2–4 weeks	Control (6) + DKD-SHED (6) + DKD-PBS (6) + DKD-BMMSC (6)	4 × 10^6^ cells in GFP-SHED or GFP-BMSCs	Tail vein injection	Decreased in the MSC group	Administration of SHED and BMSCs induced significant changes in body weight, serum cholesterol, serum triglycerides, urinary albumin, and kidney/body weight percentage in rats, suggesting potential therapeutic effects. SHED has the potential to attenuate DKD by inhibiting a process called EMT (epithelial-mesenchymal transition) that is activated by AGEs (advanced glycation end products).	[142]

*n* sample size, *N/A* not available, *DKD* diabetic kidney disease, *PMSC* placenta derived mesenchymal stem cell, *SD* Sprague-Dawley, *STZ* streptozotocin, *IP* intraperitoneal, *T1DM* type 1 diabetes mellitus, *BUN* blood urea nitrogen, *UACR* urine albumin creatinine ratio, *PD-1* programmed cell death protein 1, *PD-L1* programmed death-ligand 1, *RBG* random blood glucose, *SIRT1-PGC-1α-TFAM* sirtuin 1-peroxisome proliferator-activated receptor gamma coactivator 1-alpha-mitochondrial transcription factor A, *AMSCs* amniotic mesenchymal stem cell, *T1DRM* type 1 DKD rat model, *BGL* blood glucose levels, *ARFs* Aldose reductase family, *KIM-1* kidney injury molecule-1, *UCMSC* umbilical cord mesenchymal stem cells, *DN* diabetic nephropathy, *TECs* tubular epithelial cells, *Arg-1* arginase-1, *VCAM-1* vascular cell adhesion molecule-1, *α-SMA* α-smooth muscle actin, *TNF-α* tumor necrosis factor-α, *NGAL* neutrophil gelatinase-associated lipocalin, *ICAM-1* intercellular adhesion molecule-1, *iNOS* inducible nitric oxide synthase, *IV* intravenous, *FBG* fasting blood glucose, *HbA1c* glycated hemoglobin, *HFD* high fat diet, *HSD* high salt diet, *BM-MSC* bone marrow derived mesenchymal stem cell, *ACE2* angiotensin-converting enzyme 2, *RAS* renin-angiotensin system, *TFEB* transcription factor, *UAE* urinary albumin excretion, *ECM* extracellular matrix, *T2DM* type 2 diabetes mellitus, *TGF-β* transcription growth factor-beta, *GLUT-1* glucose transporter-1, *VEGF* vascular endothelial growth factor, *BMP-7* bone morphogenetic protein 7, *AD-MSC* adipose-derived mesenchymal stem cells, *SDT* spontaneously diabetic Torii, *SC* sham-operated-control, *EMPA* empagliflozin, *SHED* stem cells from human exfoliated deciduous teeth, *GK-rats* Goto-Kakizaki rats, *GFP* green fluorescence protein, *IL-6* interleukins-6

↓ Indicates a downward or decreasing effect

According to [117], many preclinical experimental models of DM and diabetic nephropathy have shown that exogenously administered MSCs can modulate a variety of pathophysiologic processes that contribute to the progressive renal injury and functional loss seen in DKD through paracrine-mediated actions and cell-cell interactions. The evidence suggests that intravenous or other routes of MSC administration can have beneficial effects on the kidneys in diabetes models, both directly through the transfer of MSCs and their mediators to distinct renal compartments and indirectly through the reduction of glycemia and systemic inflammation [117]. Several mediators have been identified that play a crucial role in the direct and indirect paracrine effects of MSCs in DKD. One of these mediators is indoleamine 2,3-dioxygenase (IDO), which is a potent immunomodulatory enzyme that can inhibit T-cell activation and proliferation. IDO can be induced in MSCs in response to inflammatory stimuli, and its expression can lead to the production of immunosuppressive metabolites such as kynurenine and tryptophan, which can further inhibit immune cell function. Another mediator associated with the paracrine effects of MSCs in DKD is prostaglandin E2 (PGE2). PGE2 is a lipid mediator that has been shown to play a key role in the regulation of T cell differentiation, particularly in the promotion of regulatory T cell (Treg) differentiation. In addition to IDO and PGE2, interleukin-10 (IL-10) has also been identified as an important mediator of the paracrine effects of MSCs in DKD. IL-10 is an anti-inflammatory cytokine that is produced by macrophages in response to phagocytosis of apoptotic MSCs. IL-10 can promote tissue repair and regeneration by suppressing inflammation and promoting the differentiation of pro-repair immune cells [118]. The outcomes received from DKD and DM models elucidated that MSCs can exert their potential when used as MSCs-derived conditioned medium (cocktail of cytokines, growth factors) or exosomes. The use of MSC-derived conditioned medium and exosomes offers advantages over MSCs, as it eliminates the need for isolation and expansion, reduces the risk of immune rejection, and allows for tailored administration of bioactive molecules [119].

MSCs have been found to have positive effects on the kidneys, leading to reductions in various negative processes such as glomerular size, podocyte apoptosis, glomerular matrix expansion/sclerosis, peritubular interstitial fibrosis, renal tubular epithelial cell death and dedifferentiation, tubulointerstitial fibrosis, and microvascular rarefaction. As a result, these effects are linked to decreased albuminuria (an indication of kidney damage) and stabilization of glomerular filtration rate (GFR), which is a key measure of kidney function [120].

### MSCs-derived exosomes and their role in DKD

MSCs can secret a large number of RNAs, lipids, and a variety of soluble factors packaged in extracellular vesicles (exosomes), as well as act through their paracrine function, The promising biological capacities of exosomes including biocompatibility, stability, low toxicity, and effectual transport of molecular cargos, make them a suitable candidate in cellular therapy. Studies have shown that MSCs-Exos may have beneficial effects in the treatment of neurological, respiratory, cartilage, renal, cardiac, liver diseases, bone regeneration, and cancer [143]. Compared to MSCs alone, MSCs-exosomes have demonstrated superior therapeutic and regenerative effects. Nearly, all DKD renal resident cells exhibit an autophagy disorder when diabetic [144]. Exosomes made from human urine stem cells, adipose tissue-derived MSCs, BM-MSCs, human umbilical cord MSCs, endometrial fluid, and amniotic fluid are helpful in the treatment of DKD [145]. The BM-MSCs-exosomes have potential therapeutic benefits in the treatment of DKD. Studies in animal models of DKD have shown that BM-MSC-Exos can upregulate autophagy, which is an important cellular process that helps to remove damaged proteins and organelles. This upregulation of autophagy is thought to be mediated by the inhibition of the mTOR signaling pathway, which is known to be dysregulated in DKD. The inhibition of this pathway leads to improved renal function, as evidenced by decreased levels of SCr, blood urea nitrogen (BUN), and urine albumin (UALB) in DKD mice after multiple injections of BM-MSC-Exos. The BM-MSC-Exos also have an anti-fibrotic action, they can reduce renal fibrosis, which is a key contributor to the progression of DKD. Additionally, the injections were able to reduce mesangial dilatation, a characteristic feature of diabetic kidney disease [146].

Recent research has shown that exosomes acquired from human urine stem cells can transport miR-16-5p, which is a small non-coding RNA molecule. This miRNA has displayed an important role in preventing podocyte apoptosis and inhibiting the expression of VEGF-A (vascular endothelial growth factor A), which is a key mediator of diabetic nephropathy. This inhibition of podocyte apoptosis shows potential in alleviating podocyte damage and slowing the progression of DKD. Through its intricate interactions at the molecular level, miR-16-5p emerges as a prospective facilitator for the restoration of podocyte quantities and the reinforcement of renal resilience. Through the downregulation of VEGF-A expression, a crucial contributor to the pathogenesis of diabetic nephropathy, miR-16-5p actively contributes to the modulation of podocyte behavior and the alleviation of disease-related processes. This study provides a fresh perspective for which future investigations for DN may be established upon. However, being a pre-clinical study, further investigations into the finer mechanistic details are required in future studies. In addition to this, exosomes from human urine stem cells have been found to reduce inflammation, ameliorate podocyte injury, and improve renal function in a mouse model of DKD [147]. Understanding how these molecules interact with target cells and how they influence the development of DKD could open new perspectives in the field of regenerative medicine. Studies have shown that miR-146a-5p can target the TRAF6-STAT1 signaling pathway, which is a key mediator of inflammation in the kidney. By targeting this pathway, miR-146a-5p is able to promote the polarization of M2 macrophages, which are a type of immune cell that have anti-inflammatory properties. This promotes a shift from the pro-inflammatory M1 macrophage phenotype to the anti-inflammatory M2 phenotype. Additionally, this shift in macrophage polarization leads to a suppression of renal inflammation and the restoration of renal function in preclinical models of kidney disease, such as diabetic nephropathy [148]. By carrying miRNAs that target specific pathways or genes involved in the disease, exosomes derived from MSCs may be able to modulate the behavior of renal resident cells and improve renal function. While the potential therapeutic benefits of MSC-exosomes in DKD are promising, it is important to note that additional research is required to fully understand the mechanisms underlying their effects and to evaluate the MSCs and MSC-exosomes safety and efficacy in humans.

### Use of MSCs in DKD animal models

The use of stem cell treatment has gained considerable attention as a potential strategy for treating diseases or replacing/healing damaged tissues. Studies using streptozotocin (STZ)-induced diabetes mellitus (DM) have shown that MSCs were successful in reversing hyperglycemia and preventing nephropathy in diabetic mice [105]. In the renovascular hypertension model, MSC therapy significantly reduced the progressive increase in blood vessel tension by inhibiting the renin-angiotensin system (RAS) and reducing sympathetic hyperactivity. These are two processes associated with the progression of DKD [149]. In a trial model of induced atherosclerosis, mesenchymal stem cell therapy significantly reduced dyslipidemia (abnormal lipid levels in the blood) and chronic inflammation [150]. Administration of MSCs improved vascular reactivity in individuals with heart failure and demonstrated significant potential in addressing various issues related to vascular endothelial dysfunction. MSCs have the potential to address several comorbidities associated with and contributing to the progression of DKD. MSCs are widely used for these possibilities because of their ability to modify risk factors associated with DKD. The most recent decade has seen a fast progression in cell treatment involving MSCs in clinical preliminaries, as per specialist started enlistment information from the US National Institutes of Health (https://clinicaltrials.gov/). Throughout recent years, the amount of MSC-based clinical trials have multiplied. As of July 2021, 1014 preliminary clinical studies based on MSCs were listed as completed or ongoing on Clinical Trials.gov. Similarly, as of July 2022, we searched the keyword “mesenchymal stem cells” to find the comprehensive studies count. There were 1119 studies. After applying a filter of “not yet recruiting,” it showed 69 studies; recruiting 215; enrolling by invitation 14; active not recruiting 53; suspended 16; terminated 37; withdrawn 38; unknown status 317; and COMPLETED “343” respectively. The filter of “mesenchymal stem cells” and “diabetic kidney disease” showed eight studies count with a “recruiting status: 5 studies” and “unknown studies: 3.” As of March 2023, with keywords “mesenchymal stem cells” and “diabetic kidney disease,” it showed eight studies count with a “recruiting status: 3 studies,” “enrolling by invitation: one study, and “unknown studies: 4.”

**Fig. 3:**
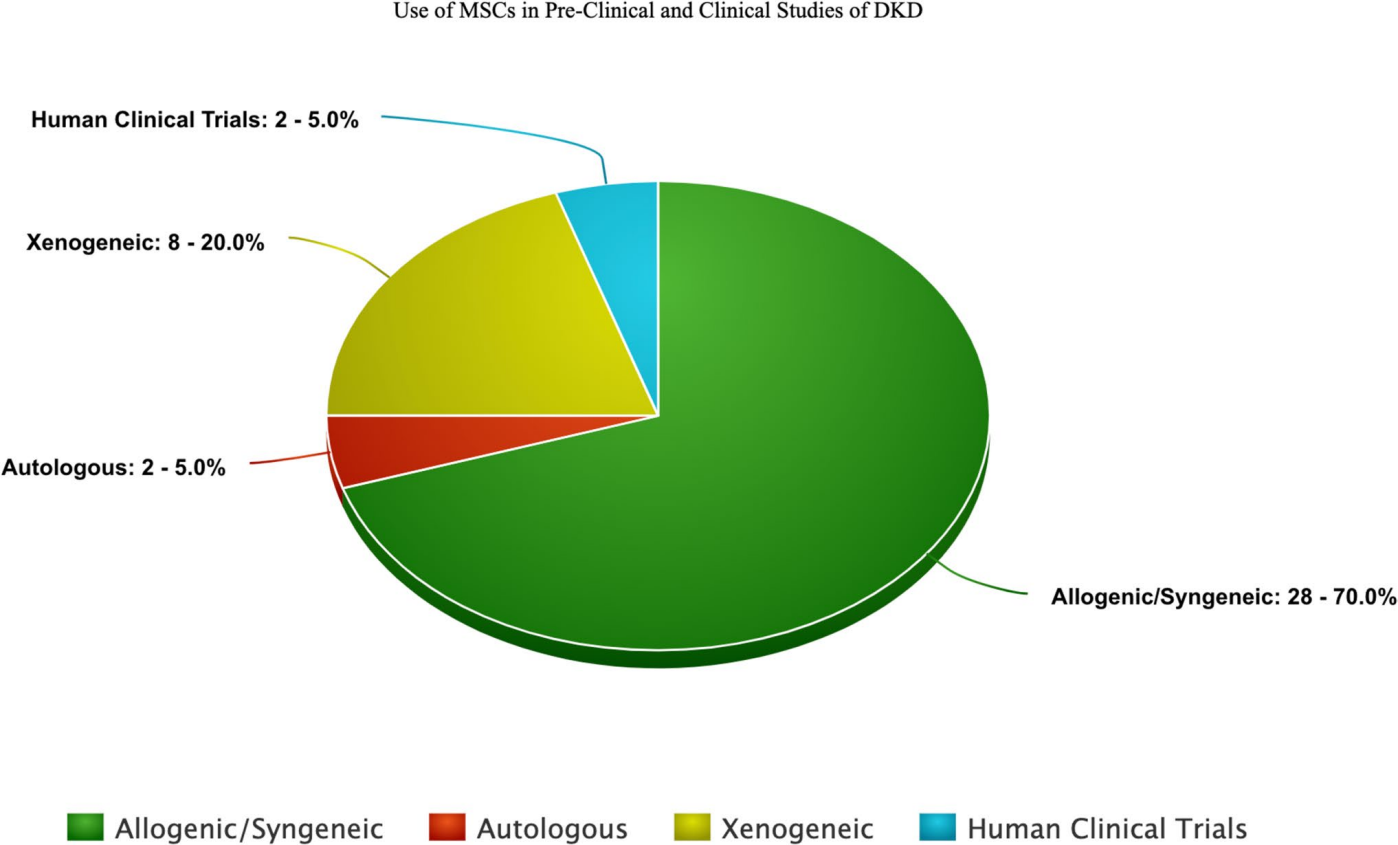
Use of MSCs in pre-clinical and clinical studies of DKD

A table has been formulated to represent preclinical trials conducted in the past decade (Table 1). The primary focus was to provide a quick insight to any researcher venturing into the DKD human clinical trials. According to the literature review, the preclinical studies were conducted on animal models like rats, mice, tree shrews, and rhesus macaques with published results in DKD [151], and only one human clinical trial was predicated [152] till 2022. Recently, a new human clinical trial (NEPHROSTROM) has been conducted and published their results as the second human clinical trial [153] (Table 4). The estimated preclinical and clinical studies count over the past few years have also been depicted in Fig. 3, based on the category/source of MSCs, indicating a dire need for extended studies on humans to predict the effects of MSCs in this diabetes-induced complication, i.e., DKD. This pie chart displays a higher number of studies in the allogenic/syngeneic category, considering the immune-privileged nature of MSCs in the renal lineage.

Furthermore, Li et al. [124] study findings elucidated that by preventing TGF-β-activated myofibroblast trans differentiation (MFT), silencing mesangial cell proliferation umpired by the P13/AKT and MAPK signal transduction pathways and driving up the matrix metalloproteinases (MMPs) concentrations in the mesangial cells, mouse UC-MSCs aids in the different roles. It tends to reduce kidney fibrosis in diabetic kidney disease or diabetic nephropathy. The role of mUC-MSCs presents an anti-fibrosis paracrine mechanism in DKD/DN. Rao et al. [142] proposed xenoplastic transplantation. Study results show that SHED (stem cells from human exfoliated deciduous teeth, which are obtained from young individuals to acquire high proliferation rate and accessibility) may become an impactful remedial resource to treat DKD, the ultimate and primary root of ESRD, as it impedes kidney damage in DKD/DN. SHED includes the prevention of hyperglycemia, lipidemia, raised protein amount in urine, extracellular matrix agglomeration, and a few segmental mesangial areas. While proposing UC-MSCs in xenoplastic transplantation [127] explained that MSCs were fine enough to reduce daily insulin intake, alleviation of HbA1c in DN subjects was also monitored. The renal functions and pathogenic abnormalities were improved, and a reduction in expression of SGLT2 on renal tubular cells was predicted. The authors of this experiment revealed no discernible alteration in the immune system of rhesus macaque DN models. In animal models of DKD, MSCs may decrease SCr, BUN, CCr, urine protein, and renal hypertrophy and increase body weight, glycemic control, and pancreatic islet activity to release insulin. MSCs can lessen renal fibrosis and the release of inflammatory mediators. Graft rejection was not reported in any of the animal experiments. Conceivably, molecular interpretations reveal that MSCs may lessen the inflammatory responses, including MCP-1 and TNF-α, as well as indications of renal fibrosis, namely Col-1, E-cadherin, and TGF-β. Conclusively, MSC therapy may 1 day be used to treat DKD. Recently studies have utilized allogeneic sources such as placenta and amniotic-derived MSCs to elucidate the impact on DKD. The results were promising. Although MSCs have shown promising results in animal models of DKD, there is still much to be learned about their mechanism of action, biodistribution, and novel and existing biomarkers and how they can be optimized for clinical translation, effect on body weight or kidney-to-body-weight ratios, and extended studies with the repeat dose of MSCs. By measuring the above-mentioned indicators, DKD management can be done at the initial stages and results of treatments like MSCs can be seen in a broader way.

In this overview, we have presented a generic overview of the preclinical and clinical trials conducted in the past based on MSC sources. This highlights the dire need to move forward with the translation of MSCs into human clinical trials for DKD. These animal models provide sufficient efficacy results and lay the foundation for investigating the role of MSCs in human subjects with DKD. Moreover, recently six clinical trials have been listed as “ongoing,” with the National Library of Medicine (NLM) listed in Table 2.

**Table 2:** Completed and in-progress clinical studies using MSC-based treatment for DKD

	**Completed**		**Registered with NLM**					
**Clinical trial ID**	NCT01843387	NCT02585622	NCT02585622	NCT04216849	NCT03288571	NCT03840343	NCT04125329	NCT04562025
**Phase**	I	Ib/IIa	I, II	I, II	I, II	I	Early phase I	N/A
**No. of subjects**	30	14	48	54	20	30	15	38
**Route**	IV	IV	IV	IV	Renal parenchyma	Intra-arterial delivery (single kidney)	Peripheral IV injection	IV
**MSCs type**	BM-MPC	ORBCEL-M	BM-MSCs	UC-MSCs	UC WJ-MSC	AD-MSCs	UC-MSCs	UC-MSCs
**Dose and frequency**	1 dose: 150, 300 × 10^6^ cells	80 × 10^6^ cells	1 dose: 80, 160, 240 × 10^6^ cells	5 doses: 1.5 × 10^6^ cells/kg	3 doses: 3 ml/kidney	2 doses: 2.5, 5 × 10^5^ cells/kg	3 doses: 1 × 10^6^ cells/kg	3 doses: 1 × 10^6^ cells
**Trial status**	COMPLETED	COMPLETED	Recruiting	Unknown	Unknown	Recruiting	Recruiting	N/A

*NLM* National Library of Medicine, *MSC* mesenchymal stem cells, *BM-MPC* bone marrow mesenchymal precursor cells, *UC* umbilical cord, *WJ* Wharton jelly, *AD-MSC* adipose-derived MSC

## Potential biomarkers for monitoring MSCs effect in DKD progression

Effective translation of cellular therapies like MSCs for diabetes and its complications in human subjects needs the identification of measurable biomarkers or factors that can serve as predictors or early indicators of favorable therapeutic response. Increased urine albumin excretion is a critical marker for progressive DKD risk, but its utility as a surrogate for future CKD/ESRD risk in clinical trials is uncertain [154]. Therefore, identifying potential biomarkers for monitoring MSC’s impact on DKD progression is crucial for successful therapeutic outcomes. Researchers have been investigating individual biomarkers or panels of biomarkers linked to an evolving understanding of the pathophysiology of DKD. Pro-inflammatory cytokines, including TNF-α, IL-6, IL-1β, and MCP-1, have emerged as possible indicators of DKD risk and severity. Multiple studies have reported elevated serum and urine TNF-α levels in individuals with DM in comparison to healthy controls, which may correlate with albuminuria status and renal function. In vivo models examining MSC therapy’s impact on renal function in rats with DKD have shown a decrease in TNF-α levels. Soluble forms of TNF-α receptors, sTNFR1 and sTNFR2, may also be potential indicators of DKD severity [155]. While the clinical significance of these biomarkers as predictors of DKD progression and complications has not been definitively established, they could potentially serve as biomarkers of the anti-inflammatory effects of MSCs. Further research is necessary to validate their clinical usefulness in predicting DKD progression and response to MSC therapy. Table 3 lists emerging biomarkers in DKD that may offer insights into the mechanisms of MSC action in animal and human models of the disease. As a result, measuring the levels of these cytokines in the blood or urine of DKD patients before and after MSC therapy may serve as a promising biomarker for assessing the effectiveness of the treatment.

**Table 3:** Potential biomarker for DKD progression

**Source**	**Biomarker**	**Key outcomes**	**Ref**
**Serum + urine** **(Individual biomarker)**	TNF-α	Progression of albuminuria in DKD and DM	[156]
	TNFR1 + 2	Predictive elevation of CKD3 T1DM and ESRD in T2DM	[157]
	Adiponectin	Progression of macroalbuminuria and ESRD	[158]
	NGAL	Progression of albuminuria	[159]
	KIM-1	Indicator of reduced eGFR in normo-albuminuria	[160]
**Serum**	FGF-23	Progression of macro-albuminuria	[161]
	FGF-21	Indicator of reduced eGFR in normo-albuminuria	[162]
	IL-6	Association between baseline eGFR levels and the effect of bone marrow mesenchymal progenitor cells (MPCs) on eGFR stabilization in phase I/II clinical trials	[120]
**Serum, urine** **(Panel biomarker)**	Peptide and metabolite panels	Increased in DKD with progression in albuminuria	[163]
	Candidate biomarker panel	Indicator of progression from CKD3	[164]

*TNF-α* tumor necrosis factor alpha, *DM* diabetes mellitus, *DKD* diabetic kidney disease, *TNFR1*+*2* TNF receptor 1 and TNF receptor 2, *CKD3* chronic kidney disease stage 3, *ESRD* end-stage renal disease, *NGAL* neutrophil gelatinase-associated lipocalin, *FGF-23* fibroblast growth factor 23, *FGF-21* fibroblast growth factor 21, *eGFR* estimated glomerular filtration rate, *KIM-1* kidney injury molecule 1, *IL-6* interleukin 6, *MPCs* mesenchymal precursor cells, *ARB* angiotensin receptor blocker

## Human clinical trials using MSCs for DKD

MSC therapy for DKD or other causes of CKD has only recently been started in clinical trials, and research is still in its infancy. Only a few clinical trials of MSC therapy in DKD and four additional trials in nondiabetic CKD were found after searching the three major clinical trial registries: WHO International Clinical Trials Registry (www.who.int/ictrp/en/), EU Clinical Trials Register (www.clinicaltrialsregister.eu/), and US National Institutes of Health ClinicalTrials.gov (www.clinicaltrials.gov). Clinical researchers are currently studying the regenerative and clinical role of MSCs in this illness. Numerous animal and in vitro studies have suggested that MSC-based therapy holds great potential for treating DKD. The outcomes of these clinical trials provide crucial information, and Table 2 lists the research foundations for the subsequent clinical studies (completed and in the process trials).

A prospective randomized controlled experiment specifying the double-blindness, dose-escalating, sequential (NCT01843387) finished in 2016 [152] was the first clinical trial. The effectiveness and adverse events were tracked in 30 randomly assigned individuals to receive their assigned arm of mesenchymal precursor cells (MPC) or a placebo.

The second clinical trial, i.e., The Novel Stromal Cell Therapy for Diabetic Kidney Disease (NEPHSTROM), a phase 1b/2a trial, has been performed at three European sites (NCT02585622). The brief summary of this study is described below. Table 4 displays the primary characteristics for both clinical trials.

**Table 4:** Intravenous administration of allogeneic MSCs in DKD patients

**MSC source**	**Age**	**DM type**	**Sample**	**Sample distribution**	**No. of injections**	**Injection method**	**Results**	**Phase**	**Adverse events (n)**	**Follow up (m)**	**Ref**
**BM-MPC**	Placebo: 74:8 ± 7:9 y, Lower dose MSCs: 70:5 ± 7:4 y Higher dose MSCs: 64:8 ± 10:1 y	T2DM	30	150 × 10^6^/kg (lower dose) or 300 × 10^6^/kg (higher dose)	Single dose	IV	↔ eGFR, albuminuria ↔ lipid profile ↔ blood pressure ↔ serum C-reactive protein, TNF-α ↓ serum IL-6	I/II	None	12	[152]
**BM-MSC**	Placebo: 54–66 y, ORBCEL-M: 66–73 y	T2DM	16	ORBCEL-M (80 × 10^6^ cells)	Single dose	IV	↔ eGFR, ↓ annual mGFR decline in groups ↔ UACR, ↔ Safety, = blood glucose; HbA1c; serum total cholesterol; serum triglycerides; and serum C-reactive protein, ↑ sTNFR1, 1NGAL, sVCAM-1, Tregs	1b/IIa	None	18	[153]

*mGFR* measured glomerulus filtration rate, *Tregs* regulatory T cells, *sTNFR1* tumor necrosis factor receptor 1,*NGAL* neutrophil gelatinase-associated lipocalin, *sVCAM-1* soluble vascular cell adhesion molecule 1, *IV* intravenous

↔ represents a no significant change or impact in either direction

↓ indicates a downward or decreasing effect

= represents stability as values or parameters remain unchanged or steady over time

↑ indicates increasing levels

## 1st clinical study using MSCs for DKD

This is the first double-blind with a control group (placebo) trial using allogeneic BM-MSCs in patients with kidney handicaps achieved by T2DM. The assigned treatment dose was administered intravenously (IV) starting the day following the pre-procedure diagnostics. Throughout the 60-week study, patients, researchers, and sponsors were kept in the dark regarding the therapy allocation. Immature BM-MSCs from a healthy paid donor who had been immunoselected with rexlemestrocel-L were retrieved. The primary goal was to overview the opportunity of immunological hypersensitivity after a singular administration of rexlemestrocel-L concerning extreme and determined safety. Regardless of the way the trial was not controlled for effectiveness, the researchers observed a relationship between isotope and Cr-based assessment at 12 weeks after an implantation procedure in patients with diabetic nephropathy. They chose to focus on the 12-week timepoint because previous studies have shown similar results at this stage in the early phase of the disease. The study measured changes in HbA1c, fasting plasma glucose, and insulin levels over time. The results show that these levels did not change significantly after some time. The study population had varying levels of hyperglycemia, with HbA1c ranging from 5.1 to 11.2%. The use of diabetes medications also varied among the study population: 20% were taking insulin, 20% were taking different oral solutions, 20% were taking both oral and insulin, and 17% were managing their diabetes only through diet. Additionally, 23.3% of the study population took only an oral medication, typically a sulfonylurea or biguanide. The study also mentions that the experts were permitted to amend the type 2 diabetes treatment for each subject as deemed appropriate during the study. The study found that the imbuements (infusions of the therapy) had positive outcomes and that the safety profile was similar for each treatment group. The researchers also evaluated potential risks associated with allogeneic cell therapy, such as allergic reactions or immunogenic reactions to human antigens from the cells. However, prior to infusion, there was no cross-matching between donor and recipient cells, and the evaluation of antibodies to the donor HLA did not prevent subjects from participating. The study also found that none of the patients developed antibodies to the donor HLA, and there was no clinically substantial increase in their % panel reactive antibodies, which are markers of an immune response. Overall, the study suggests that rexlemestrocel-L has an “immune tolerant profile,” meaning that it is not likely to trigger an immune response. However, it is important to note that this study is just one piece of evidence, and further research is necessary to fully evaluate the safety and efficacy of rexlemestrocel-L and other allogeneic cell therapies.

### Constraints in a first clinical trial

There are a few constraints in the clinical trials performed by [152]. First, the study size failed to recognize measurably massive changes in renal capability. Moreover, the chance of occurring type 1 errors cannot be precluded considering various exploratory measurable examinations without considering assortment. Moreover, regardless of whether a first-in-human review is fitting, the small cohort size (*N* = 30) cannot preclude more extraordinary well-being events that might be found more than 60 weeks following solitary implantation. Second, the study time period of 3 months to examine the adverse events of a single intervention with a 48-week follow-up time frame is not enough to predicate the disease variability and progression scale. Patients with a recent history of rapid progression of their chronic kidney disease may be more likely to see the benefits of treatment over a short period of time, compared to patients whose disease has not progressed as quickly. Third, a major limitation is that the high prevalence of albumin creatinine ratio (ACR) levels between 21 and 3000 mg g^−1^ in some patients made it difficult to evaluate changes within both groups within these boundaries. It also suggests that choosing subjects with a specific range of standard proteinuria would be more beneficial. Additionally, the study notes that because of the variability within subjects and the awareness of ongoing tests, primarily estimated inflammatory cytokines would likely require more subjects per group to detect significant changes and treatment differences over time. Finally, because of concerns about radiation exposure, additional isotopically eGFR assessments beyond 3 months (12 weeks) were not performed to support the eGFR outcomes. GFR can be misjudged or underestimated using serum creatinine-based eGFR calculations. There is a dire clinical need for developing new medications to improve renal function. It suggests that extensive research is needed to examine diabetic nephropathy, through longer and more adequately controlled studies, including occasional dosing, to evaluate the durability of the treatment. This will help determine the safety and potential resistance of allogeneic mesenchymal precursor cells and the possible efficacy signal of rexlemestrocel-L compared to placebo treatment. Aerially, it is difficult to reach any conclusion from this trial and the potential for MSC therapy in DKD remains elusive.

## 2nd clinical trial (NEPHROSTROM)

In the first cohort of the NEPHSTROM study, a phase 1b/2a clinical trial, the safety and tolerability of a single intravenous infusion of ORBCEL-M in patients with type 2 diabetes and progressive diabetic kidney disease (DKD) were explored. The intervention was well tolerated, with only one placebo-treated patient experiencing a quickly resolved infusion reaction. Importantly, the deaths of two ORBCEL-M recipients during the follow-up period were determined to be unrelated to the trial investigational product, emphasizing its acceptable safety profile. These events occurred at longer intervals following cell administration, and other medical comorbidities associated with type 2 diabetes and DKD were identified as the likely causal factors. Addressing theoretical concerns regarding the potential for transformed cells to give rise to tumors in recipients, the study highlighted that while murine MSC has demonstrated malignant transformation, there have been no reports of such events with human MSC-based therapies. Furthermore, post-mortem examinations of patients who had received allogeneic MSC for various conditions found no evidence of ectopic tissue formation or malignant tumors of MSC origin.

The study’s clinical efficacy findings revealed a significant reduction in the rate of decline in estimated glomerular filtration rate (eGFR) in the ORBCEL-M group compared to the placebo group, particularly when assessed using the CKD-EPI and MDRD equations. However, the measured GFR (mGFR) did not exhibit a significant difference between the groups. These results indicate the potential reno-protective effects of ORBCEL-M. The study also delved into the immunomodulatory effects of MSC, emphasizing their role in mediating therapeutic benefits. Recipients of ORBCEL-M displayed a sustained immunomodulatory and anti-inflammatory effect, resulting in specific changes in cell profiles. These observations suggest that ORBCEL-M could modulate aspects of progressive DKD through its influence on immune and inflammatory responses.

### Constraints in the 2nd clinical trial

Despite these positive findings, the study acknowledged several limitations, including the small sample size, the relatively short 18-month duration, and the emergence of new drug classes for DKD since the study’s initiation. Future research will need to address these limitations and explore the clinical potential of ORBCEL-M in larger and longer phase 2b studies.

## Autologous and allogeneic MSC treatments for other renal pathologies

As of March 2023, a search on “ClinicalTrials.gov” with the keywords “kidney” and “mesenchymal stem cells” was performed. The “completed” studies filter showed us eight studies. Among those studies, we have found one autologous and one allogenic study which utilized MSCs to treat CKD and lupus nephritis. A phase I (NCT02195323) autologous trial was designed to measure the tolerability and safety of BM-MSCs treatment in subjects with CKD. This trial was performed as a single arm at one center. The eligible CKD subjects (*n* = 7) were evaluated for an 18-month follow-up period. The investigational medicinal product, i.e., autologous Bone marrow-derived MSCs, were administered via the intravenous route (2 × 10^6^ cells/kg). The primary outcome measure of this investigation was safety which was analyzed with the count and experiencing adverse reactions. The secondary focus point was to lessen the ratio of eGFR. The renal histology of subjects during the follow-up time frame was compared from baseline, 1, 3, 6, 12, and 18 months. No treatment-related adverse events were observed during the experimentation phase. In addition, after the 18 months follow up, no statistical significance was observed in eGFR (*p* = 0.10) and SCr (*p* = 0.24) compared to baseline. In conclusion, subjects with CKD showed a safety profile and tolerability in the one-dose administration of autologous BM-MSCs [165]. Another phase I allogenic, interventional, non-randomized with parallel assignment trial (NCT04318600) was conducted to assess the safety, tolerability, and effectiveness profile of hA-MSCs. The primary trial endpoints were studied in patients with lupus nephritis. hA-MSCs were administered via peripheral IV to 11 lupus nephritis (LN) subjects with LN type II, III, or IV. They used 1 × 10^6^ cells/kg for the infusion, once each month for three times. Similarly, five LN subjects received a placebo (control group). All subjects were not administered IV corticosteroid pulse treatment, but they were permitted to intake oral corticosteroids and IV cyclophosphamide, dietary mycophenolate mofetil, tacrolimus, and leflunomide. The safety profile was analyzed by the number and severity of adverse reactions. The eGFR, 24-h proteinuria deviations and SLEDAI score were also observed at baseline and post-treatment for 60 weeks [166].

## Clinical trials for DKD have limitations

There has been an extreme absence of human information since just two clinical trials. There was a ton of variety and predisposition in animal tests, which made the ends uncertain. Since the exploratory animal models had short lives, preclinical examination frequently had brief perception times. The heterogeneity in this meta-examination was because of the exploratory models of diabetes (for example, animal species, the strategy used to produce diabetes, and the kind of diabetes), as well as the MSC therapy (e.g., origin, dose, recurrence, and route of administration, and time points of infusion about the onset of DKD). Future animal experiments with a standard set of strategies may explore different avenues regarding better expectations and more prominent examples. More human examinations are supposed to be directed from now on if preclinical investigations show adequate viability and safety for longer follow-ups.

## Conclusion—Strengths and limitations

The study explores the potential benefits of MSCs in treating diabetic kidney disease, which is a significant global health problem. The review considers various animal models and coordinated interventions that modulate different disease pathogenesis factors. The study highlights the paracrine mechanisms of MSCs and their ability to slow or reverse key pathogenic pathways. The authors suggest that MSC infusion in DKD might be viewed as a broad reprogramming of chronic nephrotoxic processes occurring in diabetes, potentially “retuning the clock” of renal pathologies in responsive patients. The study also suggests that MSC treatment could potentially improve glycemic control and advance other diabetic end-organ issues. The review highlights the potential for MSC treatment to be used in combination with lifestyle and pharmacological-based medicines for DKD. However, the study is limited to preclinical animal models, and the findings may not be generalizable to clinical applications in humans. The authors acknowledge the need for further research to optimize the use of MSCs and other stem/progenitor cell therapies in the treatment of diabetic kidney disease. The study highlights the need for patient selection criteria and the development/optimization of new and existing biomarkers to track DKD progression and MSC mechanism of action. The cost-effectiveness of MSC administration in DKD at different stages of severity is unknown. The authors acknowledge that MSC heterogeneity remains to be fully explored, and MSC heterogeneity in humans should be revealed in use, for instance, in single-cell RNA sequencing innovation.

## Data Availability

All supporting documentation and data could be provided.

## Abbreviations

DM: Diabetes mellitus
DKD: Diabetic kidney disease
GFR: Glomerular filtration rate
IDF: International Diabetes Federation
CKD: Chronic kidney disease
ESRF: End-stage renal failure
T2DM: Type 2 diabetes mellitus
T1DM: Type 1 diabetes mellitus
AGE: Advanced glycation end products
RIACE: Renal insufficiency and cardiovascular events
CVDs: Cardiovascular disorders
ESRD: End-stage renal disease
WHO: World Health Organization
RAAS: Renin-angiotensin-aldosterone system
TGF-β: Transforming growth factor-beta
NF-kB: Nuclear factor kappa Beta cells
ROS: Reactive oxygen species
eNOS: Endothelial nitric oxide synthase
NO: Nitric oxide
PKC: Protein kinase C
MAPK: Mitogen-associated protein kinase
TNF-α: Tumor necrosis factor
MCP-1: Monocyte chemoattractant protein-1
CSF-1: Colony-stimulating factor
MIF: Macrophage inflammatory factor
CTGF: Connective tissue growth factor
ADA: The American Diabetes Association
EASD: European Association for the Study of Diabetes
KDOQI: Kidney Disease Outcomes Quality Initiative
ACCORD: Action to Control Cardiovascular Risk in Diabetes
ACEIs: Angiotensin-converting enzyme inhibitors
ARBs: Angiotensin II receptor blockers
LDL-C: Low-density lipoprotein cholesterol
GLP1-RA: Glucagon-like peptide 1 receptor agonists
SGLT2i: Sodium-glucose cotransporter-2 inhibitors
DPPi-4: Dipeptidyl peptidase-4 inhibitors
DKA: Diabetic ketoacidosis
HSCs: Hematopoietic stem cells
MuSCs: Muscle stem cells
ISCT: International Society for Cellular Therapy
CFU: Colony forming unit
UC: Umbilical cord
AT: Adipose tissues
MSCs: Mesenchymal stem cells
UCB: Umbilical cord Blood
BM: Bone-marrow
iPSCs: Induced pluripotent stem cells
MMPs: Matrix metalloproteinases
SDF-1: Stromal cell-derived factor-1
IDO: Indoleamine 2,3-dioxygenase
PGE2: Prostaglandin E2
IL-10: Interleukin-10
SCr: Serum creatinine
BUN: Blood urea nitrogen
CCr: Creatinine clearance
Serum Cr: Serum creatinine
VEGF-A: Vascular endothelial growth factor A
NLM: National Library of Medicine
MFT: Myofibroblast transdifferentiation
SHED: Stem cells from human exfoliated deciduous teeth
DN: Diabetic nephropathy
HbA1c: Glycated hemoglobin
sTNFR1, sTNFR2: Soluble tumor necrosis factor 1 and 2
HLA: Human leukocyte antigens
eGFR: Estimated glomerulus filtration rate
LN: Lupus nephritis
IV: Intravenous
hA-MSCs: Human amniotic mesenchymal stem cells
CKD-EPI: Chronic Kidney Disease Epidemiology Collaboration
MDRD: Modification of diet in renal disease
